# Sorting signed permutations by short operations

**DOI:** 10.1186/s13015-015-0040-x

**Published:** 2015-03-25

**Authors:** Gustavo Rodrigues Galvão, Orlando Lee, Zanoni Dias

**Affiliations:** Institute of Computing, University of Campinas, Av. Albert Einstein, 1251, Campinas, 13083-852 Brazil

**Keywords:** Genome rearrangement, Short reversals, Short transpositions

## Abstract

**Background:**

During evolution, global mutations may alter the order and the orientation of the genes in a genome. Such mutations are referred to as rearrangement events, or simply operations. In unichromosomal genomes, the most common operations are reversals, which are responsible for reversing the order and orientation of a sequence of genes, and transpositions, which are responsible for switching the location of two contiguous portions of a genome. The problem of computing the minimum sequence of operations that transforms one genome into another – which is equivalent to the problem of sorting a permutation into the identity permutation – is a well-studied problem that finds application in comparative genomics. There are a number of works concerning this problem in the literature, but they generally do not take into account the length of the operations (*i.e.* the number of genes affected by the operations). Since it has been observed that short operations are prevalent in the evolution of some species, algorithms that efficiently solve this problem in the special case of short operations are of interest.

**Results:**

In this paper, we investigate the problem of sorting a signed permutation by short operations. More precisely, we study four flavors of this problem: (i) the problem of sorting a signed permutation by reversals of length at most 2; (ii) the problem of sorting a signed permutation by reversals of length at most 3; (iii) the problem of sorting a signed permutation by reversals and transpositions of length at most 2; and (iv) the problem of sorting a signed permutation by reversals and transpositions of length at most 3. We present polynomial-time solutions for problems (i) and (iii), a 5-approximation for problem (ii), and a 3-approximation for problem (iv). Moreover, we show that the expected approximation ratio of the 5-approximation algorithm is not greater than 3 for random signed permutations with more than 12 elements. Finally, we present experimental results that show that the approximation ratios of the approximation algorithms cannot be smaller than 3. In particular, this means that the approximation ratio of the 3-approximation algorithm is tight.

## Background

One of the challenges of modern science is to understand how species evolve. As evolution can be viewed as a branching process, whereby new species arise from changes occurring in living organisms, the study of the evolutionary history of a group of species is commonly made by analyzing trees whose nodes represent species and edges represent evolutionary relationships. Since these relationships are referred to as phylogeny, such trees are called phylogenetic trees.

Phylogenies can be inferred from different kinds of data, from geographic and ecological, through behavioral, morphological, and metabolic, to molecular data, such as DNA. Molecular data have the advantage of being exact and reproducible, at least within experimental error, not to mention fairly easy to obtain ([1], Chapter 12). Among the existing methods for phylogenetic reconstruction from molecular data, we focus on those referred to as distance-based methods. These methods build the phylogenetic tree corresponding to a group of species as follows. First, the evolutionary distance between each pair of species is estimated in order to generate a distance matrix *M* such that each entry *M*_*i*,*j*_ contains the evolutionary distance between species *i* and *j*. Then, the phylogenetic tree is constructed from this matrix using a specific algorithm, such as *Neighbor-Joining* [2]. Therefore, a key point of distance-based methods is how to estimate the evolutionary distance between two species.

A well-accepted approach for estimating the evolutionary distance is the genome rearrangement approach [3]. It proposes to estimate the evolutionary distance between two species using the rearrangement distance between their genomes, which is the length of the shortest sequence of genome-wide mutations, called rearrangement events, that transforms one genome into the other. Assuming genomes consist of a single linear chromosome, share the same set of genes, and contain no duplicated genes, we can represent them as permutations of integers where each integer corresponds to a gene. Besides, each integer may have a sign, + or −, indicating the gene orientation. Permutations whose elements have signs are called signed permutations and permutations whose elements do not have signs are called unsigned permutations.

By representing genomes as permutations, the problem of finding the shortest sequence of rearrangement events that transforms one genome into another can be reduced to the combinatorial problem of calculating the minimum number of operations necessary to transform one permutation into another. By algebraic properties of permutations, this problem can be equivalently stated as the problem of calculating the minimum number of operations necessary to transform one permutation into the identity permutation (+1+2…+*n*). This problem is commonly referred to as the permutation sorting problem.

Depending on the operations allowed to sort a permutation, we have a different variant of the permutation sorting problem. Reversals and transpositions are the most often considered operations for phylogenetic reconstruction. A reversal is responsible for reversing the order and flipping the signs of a sequence of elements within a permutation, while a transposition is responsible for switching the location of two contiguous portions of a permutation. The problem of sorting an unsigned permutation by reversals is an NP-hard problem [4]. It was introduced by Watterson *et al.* [5] and the best known result is due to Berman, Hannenhalli and Karpinski [6], who presented a 1.375-approximation algorithm. The problem of sorting a signed permutation by reversals was introduced by Bafna and Pevzner [7], who presented a 1.5-approximation algorithm. Hannenhalli and Pevzner [8] presented the first polynomial algorithm for this problem, which was further improved by Tannier, Bergeron and Sagot [9] to run in subquadratic time. Barder, Moret and Yan [10] showed how to determine the minimum number of reversals that sorts a signed permutation (without actually sorting) in linear time. The problem of sorting an unsigned permutation by transpositions is an NP-hard problem [11]. It was introduced by Bafna and Pevzner [12], who presented a 1.5-approximation algorithm. Later, Elias and Hartman [13] improved the approximation bound to 1.375. Variants of the permutation sorting problem which allow both reversals and transpositions are also regarded in the literature [14-16].

Simultaneously with the study of the aforementioned variants of the permutation sorting problem, some researchers have investigated variants in which bounds are imposed on the lengths of the operations. Jerrum [17] proved that the problem of sorting an unsigned permutation by reversals (or transpositions) of length 2 is solvable in polynomial time. Later, Heath and Vergara [18] considered the problem of sorting an unsigned permutation by reversals of length at most 3 and presented the best known result for it, a 2-approximation algorithm. Heath and Vergara [19,20] also considered the problem of sorting an unsigned permutation by transpositions of length at most 3 and presented a $\frac {4}{3}$-approximation algorithm. Jiang *et al.* [21] presented a (1+ *ε*)-approximation for unsigned permutations with many inversions and, more recently, Jiang *et al.* [22] also devised an $\frac {5}{4}$-approximation algorithm for sorting general unsigned permutations by transpositions of length at most 3. Finally, Vergara [23] showed that the $\frac {4}{3}$-approximation algorithm for the problem of sorting by transpositions of length at most 3 is a 2-approximation algorithm for the problem of sorting by reversals and transpositions of length at most 3.

The biological relevance of these bounded variants is grounded on the assumption that rearrangement events affecting large portions of a genome are less likely to occur. In the past, corroborating evidence has emerged, that is, separate sets of observations have shown the prevalence and significance of short reversals (*i.e.* reversals involving one or a few genes) in the evolution of bacterial genomes [24,25] and lower eukaryotes genomes [26,27]. This fact, together with the realization that signed permutations constitute a more biologically relevant model for genomes, motivated us to investigate the problem of sorting a signed permutation by short operations.

In preliminary work, Galvão and Dias [28] investigated the problem of sorting a signed permutation by reversals of length at most 3 and presented three approximation algorithms, the best one having an approximation factor of 9. In this paper, we not only present an approximation algorithm with a better approximation factor, but also consider other bounded variants. More precisely, we study four variants of the permutation sorting problem: (i) the problem of sorting a signed permutation by reversals of length at most 2, (ii) the problem of sorting a signed permutation by reversals of length at most 3, (iii) the problem of sorting a signed permutation by reversals and transpositions of length at most 2, and (iv) the problem of sorting a signed permutation by reversals and transpositions of length at most 3. We present polynomial-time solutions for problems (i) and (iii), a 5-approximation for problem (ii), and a 3-approximation for problem (iv). Moreover, we show that the expected approximation factor of the 5-approximation algorithm is not greater than 3 for random signed permutations with more than 12 elements. Finally, we present experimental results that show that the approximation factors of the approximation algorithms cannot be smaller than 3. In particular, this means that the approximation factor of the 3-approximation algorithm is tight.

## Preliminaries

In this section, we present basic definitions that are used throughout this paper, generally following [28]. Let *n* be a positive integer.

A *signed permutationπ* is a bijection of {−*n*,…,−2,−1,1,2,…,*n*} onto itself that satisfies *π*(−*i*)=−*π*(*i*) for all *i*∈{1,2,…,*n*}. The two-row notation for a signed permutation is

$$\pi = \left(\begin{array}{cccccccc} -n & \ldots & -2 & -1 & 1 & 2 & \ldots & n \\ -\pi_{n} & \ldots & -\pi_{2} & -\pi_{1} & \pi_{1} & \pi_{2} & \ldots & \pi_{n} \end{array} \right), $$

*π*_*i*_∈{1,2,…,*n*} for 1 ≤*i*≤*n*. The notation used in genome rearrangement literature, which is the one we will adopt, is the one-row notation *π*=(*π*_1_*π*_2_…*π*_*n*_). Note that we drop the mapping of the negative elements since *π*(−*i*)=−*π*(*i*) for all *i*∈{1,2,…,*n*}. By abuse of notation, we say that *π* has size *n*. The set of all signed permutations of size *n* is $S^{\pm }_{n}$.

A signed reversal *ρ*(*i*,*j*),1≤*i*≤*j*≤*n*, is an operation that transforms a signed permutation $\pi = (\pi _{1} \pi _{2} \ldots \pi _{i-1} \underline {\pi _{i} \pi _{i+1} \ldots \pi _{j-1} \pi _{j}} \pi _{j+1} \ldots \pi _{n})$ into the signed permutation $\pi \cdot \rho (i, j) = (\pi _{1} \pi _{2} \ldots \pi _{i-1} \underline {-\pi _{j} -\pi _{j-1} \ldots -\pi _{i+1} -\pi _{i}} \pi _{j+1} \ldots \pi _{n})$. A signed reversal *ρ*(*i*,*j*) is called a *signed k-reversal* if *k*=*j*−*i*+1. A signed *k*-reversal is called *short* if *k*≤3. It is called *super short* if *k*≤2.

The problem of sorting by signed short reversals consists in finding the minimum number of signed short reversals that transform a permutation $\pi \in S^{\pm }_{n}$ into the *identity permutationι*_*n*_=(+1+2…+*n*). This number is referred to as the *signed short reversal distance* of permutation *π* and it is denoted by *d*_*ssr*_(*π*). Similarly, the problem of sorting by signed super short reversals consists in finding the minimum number of signed super short reversals that transform a permutation $\pi \in S^{\pm }_{n}$ into *ι*_*n*_. This number is referred to as the *signed super short reversal distance* of permutation *π* and it is denoted by *d*_*sssr*_(*π*).

A *transpositionρ*(*i*,*j*,*k*),1≤*i*<*j*<*k*≤*n*+1, is an operation that transforms a signed permutation $\pi = (\pi _{1} \ldots \pi _{i-1} \underline {\pi _{i} \ldots \pi _{j-1}} \underline {\pi _{j} \ldots \pi _{k-1}} \pi _{k} \ldots \pi _{n})$ into the signed permutation $\pi \cdot \rho (i, j, k) = (\pi _{1} \ldots \pi _{i-1} \underline {\pi _{j} \ldots \pi _{k-1}} \underline {\pi _{i} \ldots \pi _{j-1}} \pi _{k} \ldots \pi _{n})$. A transposition *ρ*(*i*,*j*,*k*) is called an (*x*,*y*)-transposition, where *x*=*j*−*i* and *y*=*k*−*j*. An (*x*,*y*)-transposition is called *short* if *x*+*y*≤ 3. It is called *super short* if *x*+*y*=2.

The problem of sorting by signed short operations consists in finding the minimum number of signed short reversals and short transpositions that transform a permutation $\pi \in S^{\pm }_{n}$ into *ι*_*n*_. This number is referred to as the *signed short operation distance* of permutation *π* and it is denoted by *d*_*sso*_(*π*). Similarly, the problem of sorting by signed super short operations consists in finding the minimum number of signed super short reversals and super short transpositions that transform a permutation $\pi \in S^{\pm }_{n}$ into *ι*_*n*_. This number is referred to as the *signed super short operation distance* of a permutation *π* and it is denoted by *d*_*ssso*_(*π*).

We say that a pair of elements (*π*_*i*_,*π*_*j*_) of a signed permutation *π* is an *inversion* if *i*<*j* and |*π*_*i*_|>|*π*_*j*_|. The number of inversions in a signed permutation *π* is denoted by Inv(*π*).

### **Lemma1**.

Let *π* be a signed permutation. If Inv(*π*)>0, then there exists an inversion (*π*_*i*_,*π*_*j*_) such that *j*=*i*+1.

### *Proof*.

Let *π*_1_,*π*_2_,…,*π*_*i*_ be a maximal subsequence such that |*π*_1_|<|*π*_2_|<⋯<|*π*_*i*_|. Since Inv(*π*)>0, we have that *i*<*n*. So |*π*_*i*+1_|<|*π*_*i*_| and the result follows.

Let *Δ*Inv(*π*,*ρ*) denote the change in the number of inversions in a signed permutation *π* due to the application of an operation *ρ*, that is, *Δ*Inv(*π*,*ρ*)=Inv(*π*)−Inv(*π*·*ρ*). The following lemma provides bounds on the value of *Δ*Inv(*π*,*ρ*) considering that *ρ* is a short operation.

### **Lemma2**.

Let *π* be a signed permutation. Then, we have that

i) −1≤*Δ*Inv(*π*,*ρ*)≤1 *ifρ* is a super short operation,
ii) −2≤*Δ*Inv(*π*,*ρ*)≤2 *ifρ* is a short transposition, and
iii) −3≤*Δ*Inv(*π*,*ρ*)≤3 *ifρ* is a signed short reversal.

### *Proof*.

Suppose first that *ρ* is a super short operation. If *ρ* is a 1-reversal, then *Δ*Inv(*π*,*ρ*)=0. Moreover, if *ρ* is a signed 2-reversal *ρ*(*i*,*i*+1) or a (1, 1)-transposition *ρ*(*i*,*i*+1,*i*+2), then *Δ*Inv(*π*,*ρ*)=1 if (*π*_*i*_,*π*_*i*+1_) is an inversion and *Δ*Inv(*π*,*ρ*)=−1 otherwise.

Now, suppose that *ρ* is a (1, 2)-transposition *ρ*(*i*,*i*+1,*i*+2). We have that if (*π*_*i*_,*π*_*i*+1_) and (*π*_*i*_,*π*_*i*+2_) are inversions, then *Δ*Inv(*π*,*ρ*)=2. On the other hand, if (*π*_*i*_,*π*_*i*+1_) and (*π*_*i*_,*π*_*i*+2_) are not inversions, then *Δ*Inv(*π*,*ρ*)=−2. Finally, if either (*π*_*i*_,*π*_*i*+1_) or (*π*_*i*_,*π*_*i*+2_) is an inversion, then *Δ*Inv(*π*,*ρ*)=0. Note that a similar argument holds if *ρ* is a (2, 1)-transposition.

Finally, suppose that *ρ* is a signed 3-reversal *ρ*(*i*,*i*+2). We have that if |*π*_*i*_|>|*π*_*i*+1_|>|*π*_*i*+2_, then *Δ*Inv(*π*,*ρ*)=3. On the other hand, if |*π*_*i*_|<|*π*_*i*+1_|<|*π*_*i*+2_, then *Δ*Inv(*π*,*ρ*)=−3. Since in the other subcases we have that −1≤*Δ*Inv(*π*,*ρ*)≤1, the lemma follows.

## Sorting by bounded signed reversals

In this section, we present a polynomial-time solution for the problem of sorting by super short signed reversals and a 5-approximation algorithm for the problem of sorting by signed short reversals. Before we present the main results, we first introduce a useful tool for tackling these problems, the *vector diagram*. This tool was also used by Heath and Vergara [18,23] for the problem of sorting by (unsigned) short reversals.

### The vector diagram

**Figure 1:**
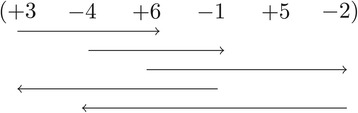
**Vector diagram.** Vector diagram of the signed permutation *π*=(+3−4+6−1+5−2). Note that Vec(*π*)=14.

For each element *π*_*i*_ of a signed permutation *π*, we define a *vectorv*(*π*_*i*_) whose length is given by |*v*(*π*_*i*_)|=||*π*_*i*_|−*i*|. If |*v*(*π*_*i*_)|>0, the vector *v*(*π*_*i*_) has a direction indicated by the sign of |*π*_*i*_|−*i*. The vector *v*(*π*_*i*_) is a *right vector* if |*π*_*i*_|−*i*>0 while it is a *left vector* if |*π*_*i*_|−*i*<0. If the length of *v*(*π*_*i*_) is zero, then *v*(*π*_*i*_) is said to be a *positive zero vector* if *π*_*i*_=*i* and a *negative zero vector* if *π*_*i*_=−*i*. A vector diagram *V*_*π*_ of *π* is the set of vectors of the elements of *π*. The sum of the lengths of all the vectors in *V*_*π*_ is denoted by Vec(*π*). See Figure 1 for an example.

Two elements *π*_*i*_ and *π*_*j*_,*i*<*j*, of a signed permutation *π* are said to be *vector-opposite* if the vectors *v*(*π*_*i*_) and *v*(*π*_*j*_) differ in direction, |*v*(*π*_*i*_)|≥*j*−*i*, and |*v*(*π*_*j*_)|≥*j*−*i*. Besides, they are said to be *m-vector-opposite* if *j*−*i*=*m*. Note that m specifies the distance between vector-opposite elements. For instance, in Figure 1 the elements *π*_2_=−4 and *π*_4_=−1 are 2-vector-opposite elements.

#### **Lemma3**.

Let *π* be a signed permutation. If Inv(*π*)>0, then *π* contains at least a pair of vector-opposite elements.

#### *Proof*.

We say that an element *π*_*e*_ in *π* is out-of-place if |*π*_*e*_|≠*e*. Note that there must exist out-of-place elements in *π* if Inv(*π*)>0. Among all out-of-place elements in *π*, let *π*_*i*_ be the one with the greatest absolute value. We first show by contradiction that *v*(*π*_*i*_) is a right vector. Suppose *v*(*π*_*i*_) is a left vector, that is, |*π*_*i*_|−*i*<0. Then the element *π*_*k*_ such that |*π*_*k*_|=*i* is an out-of-place element with absolute value greater than |*π*_*i*_|, a contradiction.

Now since there is at least one right vector in *V*_*π*_, there exists a rightmost right vector in *V*_*π*_, that is, a right vector *v*(*π*_*i*_) such that i is as large as possible. The element *π*_*k*_ such that *k*=|*π*_*i*_| is out-of-place since |*π*_*k*_|≠*k*. The vector *v*(*π*_*k*_) is therefore a left vector as it occurs to the right of *v*(*π*_*i*_), the rightmost right vector. Consider the elements *π*_*i*+1_,*π*_*i*+2_,…,*π*_*k*_. At least one of these elements corresponds to a left vector. Select the leftmost left vector from these elements, that is, select the vector *v*(*π*_*j*_) such that *i*+1≤*j*≤*k* and *j* is as small as possible.

We claim that *π*_*i*_ and *π*_*j*_ are vector-opposite elements. Since |*v*(*π*_*i*_)|=*k*≥*j*, all that remains to be shown is that |*v*(*π*_*j*_)|≤*i*. In other words, we need to show that the correct position of element *π*_*j*_ does not occur to the right of position i. For a contradiction, suppose this is the case. Then the element *π*_*t*_ such that *t*=|*π*_*j*_| is out-of-place and therefore *v*(*π*_*t*_) is either a right or left vector. It is not a right vector since it occurs on the right of *v*(*π*_*i*_), the rightmost right vector. It is not a left vector since it occurs on the left of *v*(*π*_*j*_), the leftmost left vector from a set that includes *v*(*π*_*t*_). Then we have a contradiction since we have found an out-of-place element that corresponds to a zero vector. The lemma follows.

#### **Lemma4**.

Let $\pi \in S^{\pm }_{n}$ be a signed permutation such that Inv(*π*)>0 and let *π*_*i*_ and *π*_*j*_ be m-vector-opposite elements. Moreover, let $\pi ^{\prime } \in S^{\pm }_{n}$ be a signed permutation such that $|\pi ^{\prime }_{i}| = |\pi _{j}|, |\pi ^{\prime }_{j}| = |\pi _{i}|$, and $|\pi ^{\prime }_{k}| = |\pi _{k}|$ for all *k*∉{*i*,*j*}. Then Vec(*π*)−Vec(*π*^′^)=2*m*.

#### *Proof*.

We have that

$$\begin{array}{rcl} {} \text{Vec}(\pi) - \text{Vec}\left(\pi^{\prime}\right) & = & \sum_{k=1}^{n}\left(\left|v\left(\pi_{k}\right)\right| - \left|v\left(\pi^{\prime}_{k}\right)\right|\right) \\ & = & \left|v\left(\pi_{i}\right)\right| - \left|v\left(\pi^{\prime}_{i}\right)\right| + \left|v\left(\pi_{j}\right)\right| \\ &&- \left|v\left(\pi^{\prime}_{j}\right)\right| \\ & = & m + m \\ & = & 2m, \end{array} $$

and therefore the lemma follows.

Let *Δ*Vec(*π*,*ρ*) denote the change in the sum of the lengths of all the vectors in *V*_*π*_ due to the application of a signed reversal *ρ*, that is, *Δ*Vec(*π*,*ρ*)=Vec(*π*)−Vec(*π*·*ρ*). The following lemma provides bounds on the value of *Δ*Vec(*π*,*ρ*) considering that *ρ* is a signed short reversal.

#### **Lemma5**.

Let *π* be a signed permutation. Then, we have that

i) *Δ*Vec(*π*,*ρ*)=0 if *ρ* is a signed 1-reversal,
ii) −2≤*Δ*Vec(*π*,*ρ*)≤2 if *ρ* is a signed 2-reversal, and
iii) −4≤*Δ*Vec(*π*,*ρ*)≤4 if *ρ* is a signed 3-reversal.

#### *Proof*.

Suppose first that *ρ* is a signed 1-reversal *ρ*(*i*,*i*). In this case, *ρ* does not affect the length of the vector *v*(*π*_*i*_), therefore *Δ*Vec(*π*,*ρ*)=0.

Now, suppose that *ρ* is a signed 2-reversal *ρ*(*i*,*i*+1). If the elements *π*_*i*_ and *π*_*i*+1_ are 1-vector-opposite, then *Δ*Vec(*π*,*ρ*)=2. On the other hand, if *v*(*π*_*i*_) is a zero or a left vector and *v*(*π*_*i*+1_) is a zero or a right vector, then *Δ*Vec(*π*,*ρ*)=−2. Note that *Δ*Vec(*π*,*ρ*) cannot be greater than 2 and cannot be less than -2 because *ρ*(*i*,*i*+1) can increase or decrease the length of *v*(*π*_*i*_) and *v*(*π*_*i*+1_) by just one unit.

Finally, suppose that *ρ* is a signed 3-reversal *ρ*(*i*,*i*+2). Note that *ρ* does not affect the length of the vector *v*(*π*_*i*+1_). Now, if the elements *π*_*i*_ and *π*_*i*+2_ are 2-vector-opposite, then *Δ*Vec(*π*,*ρ*)=4. On the other hand, if *v*(*π*_*i*_) is a zero or a left vector and *v*(*π*_*i*+2_) is a zero or a right vector, then *Δ*Vec(*π*,*ρ*)=−4. Note that *Δ*Vec(*π*,*ρ*) cannot be greater than 4 and cannot be less than −4 because *ρ*(*i*,*i*+2) can increase or decrease the length of *v*(*π*_*i*_) and *v*(*π*_*i*+2_) by just two units.

### Sorting by signed super short reversals

From the proof of Lemma 2, we have that a signed 1-reversal does not change the number of inversions in a signed permutation and a signed 2-reversal can eliminate at most one inversion. This means that, for sorting a signed permutation *π*, we have to apply Inv(*π*) signed 2-reversals plus a given number of signed 1-reversals in order to flip the signs of the remaining negative elements. The question is: how many signed 1-reversals do we have to apply?

Intuitively, if an element *π*_*i*_ is in t distinct pairs of inversions in a signed permutation *π*, then its sign will be flipped t times, one time per signed 2-reversal applied. Therefore, if *π*_*i*_ is negative and t is even, then *π*_*i*_ will remain negative after we apply the t signed 2-reversals. The same is true when *π*_*i*_ is positive and t is odd. We can make use of the vector diagram in order to capture this intuition formally.

Let $V^{even^{-}}_{\pi }$ be a subset of *V*_*π*_ such that $V^{even^{-}}_{\pi } = \{v(\pi _{i}) : \pi _{i} < 0$ and |*v*(*π*_*i*_)| is even} and let $V^{odd^{+}}_{\pi }$ be a subset of *V*_*π*_ such that $V^{odd^{+}}_{\pi } = \{v(\pi _{i}) : \pi _{i} > 0$ and |*v*(*π*_*i*_)| is odd}. The elements of a signed permutation *π* whose vectors belong to either $V^{even^{-}}_{\pi }$ or $V^{odd^{+}}_{\pi }$ are precisely the elements which will be negative after we apply the Inv(*π*) signed 2-reversals (Lemma 6). Using this fact, we can obtain an exact formula for the signed super short reversal distance of a signed permutation *π* (Theorem 1).

#### **Lemma6**.

Let *π* be a signed permutation and let *π*^′^=*π*·*ρ*(*i*,*i*+1). Then, we have that $|V^{even^{-}}_{\pi ^{\prime }}| + |V^{odd^{+}}_{\pi ^{\prime }}| = |V^{even^{-}}_{\pi }| + |V^{odd^{+}}_{\pi }|$.

#### *Proof*.

The signed 2-reversal *ρ*(*i*,*i*+1) changes the signs of *π*_*i*_ and *π*_*i*+1_ along with the parities of |*v*(*π*_*i*_)| and |*v*(*π*_*i*+1_)|. For this reason, if *π*_*i*_(or *π*_*i*+1_) belongs to either $V^{even^{-}}_{\pi }$ or $V^{odd^{+}}_{\pi }$, then $\pi ^{\prime }_{i+1} = -\pi _{i}$ (or $\pi ^{\prime }_{i} = -\pi _{i+1})$ belongs to either $V^{even^{-}}_{\pi ^{\prime }}$ or $V^{odd^{+}}_{\pi ^{\prime }}$. On the other hand, if *π*_*i*_(or *π*_*i*+1_) does not belong to neither $V^{even^{-}}_{\pi }$ nor $V^{odd^{+}}_{\pi }$, then $\pi ^{\prime }_{i+1} = -\pi _{i}$ (or $\pi ^{\prime }_{i} = -\pi _{i+1}$) does not belong to either $V^{even^{-}}_{\pi ^{\prime }}$ or $V^{odd^{+}}_{\pi ^{\prime }}$. Therefore the lemma follows.

#### **Lemma7**.

Let *π* be a signed permutation. Then, we have that $d_{\textit {sssr}}(\pi) \leq \text {Inv}(\pi) + |V^{even^{-}}_{\pi }| + |V^{odd^{+}}_{\pi }|$.

#### *Proof*.

It suffices to prove that it is always possible to apply signed super short reversals on *π*≠*ι*_*n*_ in such a way that the resulting permutation *π*^′^ satisfies

(1) $$\begin{array}{@{}rcl@{}} \text{Inv}(\pi^{\prime}) &+& |V^{even^{-}}_{\pi^{\prime}}| + |V^{odd^{+}}_{\pi^{\prime}}| \leq \text{Inv}(\pi) + |V^{even^{-}}_{\pi}|\\ &+& |V^{odd^{+}}_{\pi}| - 1. \end{array} $$

If Inv(*π*)=0, then |*v*(*π*_*i*_)|=0 for every *π*_*i*_ of *π*. This means that $|V^{odd^{+}}_{\pi }| = 0$, and therefore we can sort *π* with $|V^{even^{-}}_{\pi }|$ signed 1-reversals and (1) holds.

If Inv(*π*)>0, then there exists a signed 2-reversal *ρ*(*i*,*i*+1) that removes an inversion in *π* (Lemma 1). So, apply such signed 2-reversal on *π* and let *π*^′^ denote the resulting permutation. We have that Inv(*π*^′^)=Inv(*π*)−1. Moreover, we have that $\lvert V^{even^{-}}_{\pi ^{\prime }} \lvert + \lvert V^{odd^{+}}_{\pi ^{\prime }} \lvert = \lvert V^{even^{-}}_{\pi } \lvert + \lvert V^{odd^{+}}_{\pi } \lvert $ (Lemma 6). Summing both equalities we obtain (1), therefore the lemma follows.

#### **Lemma8**.

Let *π* be a signed permutation. Then, we have that $d_{\textit {sssr}}(\pi) \geq \text {Inv}(\pi) + \lvert V^{even^{-}}_{\pi } \lvert + \lvert V^{odd^{+}}_{\pi } \lvert $.

#### *Proof*.

It suffices to prove that if we apply an arbitrary signed super short reversal on *π*, then the resulting permutation *π*^′^ satisfies

(2) $$\begin{array}{@{}rcl@{}} \text{Inv}(\pi^{\prime}) &+& |V^{even^{-}}_{\pi^{\prime}}| + |V^{odd^{+}}_{\pi^{\prime}}| \geq \text{Inv}(\pi) + |V^{even^{-}}_{\pi}|\\ &+& |V^{odd^{+}}_{\pi}| - 1. \end{array} $$

Suppose first that we apply a signed 1-reversal *ρ*(*i*,*i*) on *π* and let *π*^′^ denote the resulting permutation. We have that Inv(*π*^′^)=Inv(*π*). Moreover, since the sign of *π*_*i*_ is flipped without changing the parity of |*v*(*π*_*i*_)|, we have that $\lvert V^{even^{-}}_{\pi ^{\prime }} \lvert + \lvert V^{odd^{+}}_{\pi ^{\prime }} \rvert \geq \lvert V^{even^{-}}_{\pi } \rvert + \lvert V^{odd^{+}}_{\pi }\rvert - 1$. Summing the previous equality with this inequality we obtain (2).

Now, suppose that we apply a signed 2-reversal *ρ*(*i*,*i*+1) on *π* and let *π*^′^ denote the resulting permutation. We have that $\lvert V^{even^{-}}_{\pi ^{\prime }} \lvert + \lvert V^{odd^{+}}_{\pi ^{\prime }} \lvert = \lvert V^{even^{-}}_{\pi } \lvert + \lvert V^{odd^{+}}_{\pi } \lvert $ (Lemma 6). Moreover, since a signed 2-reversal can remove at most one inversion, we have that Inv(*π*^′^)≥Inv(*π*)−1. Summing the previous equality with this inequality we obtain (2). Therefore the lemma follows.

#### **Theorem1**.

Let *π* be a signed permutation. Then, we have that $d_{\textit {sssr}}(\pi) = \text {Inv}(\pi) + \lvert V^{even^{-}}_{\pi } \lvert + \lvert V^{odd^{+}}_{\pi } \lvert $.

#### *Proof*.

Immediate from Lemmas 7 and 8.

From the proof of Lemma 7, we can derive the following optimal algorithm for sorting a signed permutation by signed super short reversals. First, perform signed 2-reversals on the inversions until the permutation has no inversions. Then, perform signed 1-reversals on the negative elements until the permutation has no negative elements. Since a signed permutation $\pi \in S^{\pm }_{n}$ can have at most $\binom {n}{2}$ inversions and at most n negative elements, we have that this algorithm runs in *O*(*n*^2^) time. We remark that the value of *d*_*sssr*_(*π*) can be computed in $O(n\, \sqrt {\log {n}})$ time because computing $\lvert V^{even^{-}}_{\pi } \lvert + \lvert V^{odd^{+}}_{\pi }\lvert $ takes *O*(*n*) time and computing Inv(*π*) takes $O(n\,\sqrt {\log {n}})$ time [29].

### Sorting by signed short reversals

A trivial algorithm for the problem of sorting by signed short reversals is the optimal algorithm for the problem of sorting by signed super short reversals. From the lower bound of Lemma 9, it follows that this trivial algorithm is a 6-approximation algorithm. Moreover, we have that this approximation bound is tight. For instance, we need 6 signed super short reversals for sorting the signed permutation (−3−2−1), but one signed 3-reversal is sufficient for sorting it.

#### **Lemma9**.

Let *π* be a signed permutation. Then, we have that $d_{\textit {ssr}}(\pi) \geq \frac {\text {Inv}(\pi) + |V^{-}_{\pi }| + |V^{+}_{\pi }|}{6}$.

#### *Proof*.

It suffices to prove that if we apply an arbitrary signed short reversal on *π*, then the resulting permutation *π*^′^ satisfies

(3) $$\begin{array}{@{}rcl@{}} \text{Inv}(\pi^{\prime}) &+& |V^{even^{-}}_{\pi^{\prime}}| + |V^{odd^{+}}_{\pi^{\prime}}| \geq \text{Inv}(\pi) + |V^{even^{-}}_{\pi}|\\ &+& |V^{odd^{+}}_{\pi}| - 6. \end{array} $$

From the proof of Lemma 8, we have that (3) holds when we apply a signed super short reversal on *π*. So, suppose that we apply the signed 3-reversal *ρ*(*i*,*i*+2) on *π* and let *π*^′^ denote the resulting permutation. We have that Inv(*π*^′^)≥Inv(*π*)−3. Moreover, we have that $\lvert V^{even^{-}}_{\pi ^{\prime }} \lvert + \lvert V^{odd^{+}}_{\pi ^{\prime }} \rvert \geq \lvert V^{even^{-}}_{\pi } \rvert + \lvert V^{odd^{+}}_{\pi } \rvert - 3$. Summing both inequalities we obtain (3), and the lemma follows.

Let $V^{odd}_{\pi }$ be a subset of *V*_*π*_ such that $V^{odd}_{\pi } = \{v(\pi _{i}) : |v(\pi _{i})|\ \text {is odd}\}$ and let $V^{0-}_{\pi }$ be a subset of *V*_*π*_ such that $V^{0^{-}}_{\pi } = \{v(\pi _{i}) : v(\pi _{i}) ~~\text {is a negative zero vector}\}$. By using these two subsets of *V*_*π*_, we can obtain better bounds on the signed short reversal distance of a signed permutation *π* (Lemmas 11 and 12). These bounds lead to a 5-approximation for the problem of sorting by signed short reversals (Theorem 2). We note that the upper bound given in Lemma 11 relies on the fact that it is always possible to switch the positions of a pair of *m*-vector-opposite elements (without affecting the elements between them) applying *m* signed short reversals (Lemma 10).

#### **Lemma10**.

Let $\pi \in S^{\pm }_{n}$ be a signed permutation such that Inv (*π*)>0 and let *π*_*i*_ and *π*_*j*_ be *m*-vector-opposite elements. It is possible to transform *π* into $\pi ^{\prime } \in S^{\pm }_{n}$ such that $|\pi ^{\prime }_{i}| = |\pi _{j}|, |\pi ^{\prime }_{j}| = |\pi _{i}$, and $|\pi ^{\prime }_{k}| = |\pi _{k}|$ for all *k*∉{*i*,*j*} applying *d* signed short reversals, where

$$d = \left\{ \begin{array}{l l} m - 1 & \quad \text{if}~~ m~~ \text{is even},\\ m & \quad \text{if}~~ m ~~\text{is odd.} \end{array} \right. $$

#### *Proof*.

We have two cases to consider:

- *m* is even. In this case, we can transform *π* into a signed permutation $\pi ^{\prime } \in S^{\pm }_{n}$ such that $|\pi ^{\prime }_{i}| = |\pi _{j}|, |\pi ^{\prime }_{j}| = |\pi _{i}|, \pi ^{\prime }_{j-1} = -\pi _{j-1}$, and $\pi ^{\prime }_{k} = \pi _{k}$ for all *k*∉{*i*,*j*−1,*j*} applying the sequence of signed short reversals *ρ*(*i*,*i*+2),*ρ*(*i*+2,*i*+4),…,*ρ*(*j*−4,*j*−2)),*ρ*(*j*−2,*j*)),*ρ*(*j*−4,*j*−2),…,*ρ*(*i*,*i*+2). Therefore, to transform *π* into *π*^′^, we can apply m-1 signed 3-reversals.
- m is odd. In this case, we can transform *π* into a signed permutation $\pi ^{\prime } \in S^{\pm }_{n}$ such that $|\pi ^{\prime }_{i}| = |\pi _{j}|, |\pi ^{\prime }_{j}| = |\pi _{i}|$, and $\pi ^{\prime }_{k} = \pi _{k}$ for all *k*∉{*i*,*j*} applying the sequence of signed short reversals *ρ*(*i*,*i*+2),*ρ*(*i*+2,*i*+4),…,*ρ*(*j*−3,*j*−1),*ρ*(*j*−1,*j*),*ρ*(*j*−3,*j*−1),…,*ρ*(*i*,*i*+2). Therefore, to transform *π* into *π*^′^, we can apply m-1 signed 3-reversals and one signed 2-reversal, totalizing m signed short reversals.

Since in both cases we can transform *π* into *π*^′^ applying $2\lceil \frac {m}{2}\rceil - 1$, the lemma follows.

#### **Lemma11**.

Let *π* be a signed permutation. Then, we have that $d_{\textit {ssr}}(\pi) \leq \text {Vec}(\pi) + |V^{odd}_{\pi }| + |V^{0^{-}}_{\pi }|$.

#### *Proof*.

It suffices to prove that it is always possible to apply a sequence of *t*>0 signed short reversals on *π*≠*ι*_*n*_ in such a way that the resulting permutation *π*^′^ satisfies

(4) $$\begin{array}{@{}rcl@{}} \text{Vec}(\pi^{\prime}) &+& |V^{odd}_{\pi^{\prime}}| + |V^{0^{-}}_{\pi^{\prime}}| \leq \text{Vec}(\pi) + |V^{odd}_{\pi}|\\ &+& |V^{0^{-}}_{\pi}| - t. \end{array} $$

If Vec(*π*)=0, then |*v*(*π*_*i*_)| =0 for every *π*_*i*_ in *π*. This means that $|V^{odd}_{\pi }| = 0$. Therefore we can sort *π* with $|V^{0^{-}}_{\pi }|$ signed 1-reversals and (4) holds.

If Vec(*π*)>0, then *π* contains at least one pair of vector-opposite elements (Lemma 3). Let *π*_*i*_ and *π*_*j*_,*i*<*j*, be m-vector-opposite elements. Now, suppose that we apply the d signed reversals described in Lemma 10 on *π* and let *π*^′^ denote the resulting permutation. We will show that the application of this sequence of signed short reversals results in an average decrease in

$${\fontsize{8.9}{6}{\begin{array}{rcl} {} \Delta(\pi, \pi^{\prime}) & \,=\,\! & \text{Vec}(\pi) \,+\, |V^{odd}_{\pi}| \,+\, |V^{0^{-}}_{\pi}| \,-\, \left(\text{Vec}(\pi^{\prime}) \,+\, |V^{odd}_{\pi^{\prime}}| \,+\, |V^{0^{-}}_{\pi^{\prime}}\!|\right) \\ & \,=\, & 2m \,+\, \left(|V^{odd}_{\pi}| \,-\, |V^{odd}_{\pi^{\prime}}|\right) \,+\, \left(|V^{0^{-}}_{\pi}| \,-\, |V^{0^{-}}_{\pi^{\prime}}|\right) \end{array}}} $$

of at least 1 unit per signed short reversal. In other words, we need to show that $\frac {\Delta (\pi, \pi ^{\prime })}{d} \geq 1$.

In order the evaluate the value of *Δ*(*π*,*π*^′^), we divide our analysis in two cases:

- *m* is even. In this case, we have that the parities of the lengths of the vectors do not change, therefore $|V^{odd}_{\pi }| - |V^{odd}_{\pi ^{\prime }}| = 0$. In order to evaluate the value of $|V^{0^{-}}_{\pi }| - |V^{0^{-}}_{\pi ^{\prime }}|$, we further divide our analysis into three subcases:
  i) |*v*(*π*_*i*_)| and |*v*(*π*_*j*_)| are even. In this subcase, we have that the vectors *v*(*π*_*i*_),*v*(*π*_*j*−1_), and *v*(*π*_*j*_) may become negative zero vectors, therefore $|V^{0^{-}}_{\pi }| - |V^{0^{-}}_{\pi ^{\prime }}| \geq -3$. This means that *Δ*(*π*,*π*^′^)≥2*m*−3.
  ii) |*v*(*π*_*i*_)| and |*v*(*π*_*j*_)| have distinct parities. In this subcase, we have that the vector *v*(*π*_*j*−1_) and one of the vectors *v*(*π*_*i*_) and *v*(*π*_*j*_) (precisely the one whose length is even) may become negative zero vectors, therefore $|V^{0^{-}}_{\pi }| - |V^{0^{-}}_{\pi ^{\prime }}| \geq -2$. This means that *Δ*(*π*,*π*^′^)≥2*m*−2.
  iii) |*v*(*π*_*i*_)| and |*v*(*π*_*j*_)| are odd. In this subcase, we have that none of the vectors *v*(*π*_*i*_) and *v*(*π*_*j*_) can become a negative zero vector, but the vector *v*(*π*_*j*−1_) can. Therefore $|V^{0^{-}}_{\pi }| - |V^{0^{-}}_{\pi ^{\prime }}| \geq -1$. This means that *Δ*(*π*,*π*^′^)≥2*m*−1.
- *m* is odd. In this case, we further divide our analysis into three subcases:
  i) |*v*(*π*_*i*_)| and |*v*(*π*_*j*_)| are even. In this subcase, we have that none of the vectors *v*(*π*_*i*_) and *v*(*π*_*j*_) can become a negative zero vector, therefore $|V^{0^{-}}_{\pi }| - |V^{0^{-}}_{\pi ^{\prime }}| = 0$. Moreover, |*v*(*π*_*i*_)| and |*v*(*π*_*j*_)| become odd, therefore $|V^{odd}_{\pi } - |V^{odd}_{\pi ^{\prime }}| = -2$. This means that *Δ*(*π*,*π*^′^)=2*m*−2.
  ii) |*v*(*π*_*i*_)| and |*v*(*π*_*j*_)| have distinct parities. In this subcase, we have that the parities of the lengths of the vectors *v*(*π*_*i*_) and *v*(*π*_*j*_) are switched, therefore $|V^{odd}_{\pi }| - |V^{odd}_{\pi ^{\prime }}| = 0$. Moreover, one of the vectors *v*(*π*_*i*_) and *v*(*π*_*j*_) (precisely the one whose length is odd) may become a negative zero vector, therefore $|V^{0^{-}}_{\pi }| - |V^{0^{-}}_{\pi ^{\prime }}| \geq -1$. This means that *Δ*(*π*,*π*^′^)≥2*m*−1.
  iii) |*v*(*π*_*i*_)| and |*v*(*π*_*j*_)| are odd. In this subcase, we have that |*v*(*π*_*i*_)| and |*v*(*π*_*j*_)| become even, therefore $|V^{odd}_{\pi }| - |V^{odd}_{\pi ^{\prime }}| = 2$. On the other hand, we have that the vectors *v*(*π*_*i*_) and *v*(*π*_*j*_) may become negative zero vectors, therefore $|V^{0^{-}}_{\pi }| - |V^{0^{-}}_{\pi ^{\prime }}| \geq -2$. This means that *Δ*(*π*,*π*^′^)≥2*m*.

Note that the only subcase in which we have $\frac {\Delta (\pi, \pi ^{\prime })}{d} < 1$ is subcase (b.i), precisely when *m*=1. So, assume that we have no choice other than selecting a pair of 1-vector-opposite elements *π*_*i*_ and *π*_*j*_ such that |*v*(*π*_*i*_)| and |*v*(*π*_*j*_)| are even. We will show that it is still possible to apply a sequence of signed short reversals on *π* in such a way that (4) holds.

Let *v*(*π*_*i*_) be the rightmost right vector of *π*, that is, i is the largest integer for which *v*(*π*_*i*_) is a right vector. As shown in the proof of Lemma 3, there exists an element *π*_*j*_,*j*>*i*, such that *π*_*i*_ and *π*_*j*_ form a pair of vector-opposite elements. Combining this fact with our initial assumption, we can conclude that *j*=*i*+1.

Now, suppose that we apply the signed short reversal *ρ*(*i*,*i*+1) on *π* and let *π*^′^ denote the resulting permutation. From our previous case-by-case analysis, we have that *Δ*(*π*,*π*^′^)=0. Moreover, we have that $v(\pi ^{\prime }_{i+1})$ is the rightmost right vector of *π*^′^. Therefore, there exists an element $\pi ^{\prime }_{k}, k > i + 1$, such that $\pi ^{\prime }_{i+1}$ and $\pi ^{\prime }_{k}$ form a pair of m-vector-opposite elements, as shown in the proof of Lemma 3. This means that we can apply the d short signed reversals described in Lemma 10 on *π*^′^, obtaining permutation *π*^′′^. Given that $|v(\pi ^{\prime }_{i+1})|$ is odd, we can conclude from our previous case-by-case analysis that *Δ*(*π*^′^,*π*^′′^)≥2*m*−1 if *m* is odd and *Δ*(*π*^′^,*π*^′′^)≥2*m*−2 if *m* is even. Hence, the average decrease in *Δ*(*π*,*π*^′′^) is of at least $\frac {2m-1}{m+1}$ units per signed short reversal if m is odd and of at least $\frac {2m-2}{m}$ units per signed short reversal if m is even.

Note that $\frac {2m-1}{m+1} < 1$ when *m*=1, but in this case we show that the average decrease in *Δ*(*π*,*π*^′′^) is of at least 1 unit per signed short reversal. We have two cases to consider:

1. $|v(\pi ^{\prime }_{k})|$ is odd. In this case, we have that *Δ*(*π*^′^,*π*^′′^)≥2, therefore the average decrease in *Δ*(*π*,*π*^′′^) is of at least 1 unit per signed short reversal.
2. $|v(\pi ^{\prime }_{k})|$ is even. We show that this case cannot happen. For the sake of contradiction, assume that $|v(\pi ^{\prime }_{k})|$ is even. Then, we have that $|v(\pi ^{\prime }_{k})| \geq 2$. Besides, since *m*=1, we have that *k*=*i*+2. These two facts imply that *π*_*i*_ and *π*_*i*+2_ are 2-vector-opposite elements, but it contradicts our initial hypothesis that we had no choice other than selecting a pair of 1-vector-opposite elements.

Since it always possible to apply a sequence of *t* signed short reversals on *π* in such a way that the resulting permutation *π*^′^ satisfies (4), the lemma follows.

#### **Lemma12**.

Let *π* be a signed permutation. Then, we have that $d_{\textit {ssr}}(\pi) \geq \frac {\text {Vec}(\pi) + |V^{odd}_{\pi }| + |V^{0^{-}}_{\pi }|}{5}$.

#### *Proof*.

It suffices to prove that if we apply an arbitrary signed short reversal on *π*, then the resulting permutation *π*^′^ satisfies

(5) $$\begin{array}{@{}rcl@{}} \text{Vec}(\pi^{\prime}) &+& \left|V^{odd}_{\pi^{\prime}}\right| + \left|V^{0^{-}}_{\pi^{\prime}}\right| \geq \text{Vec}(\pi) + \left|V^{odd}_{\pi}\right| \\ &+& \left|V^{0^{-}}_{\pi}\right| - 5. \end{array} $$

Suppose first that we apply a signed 1-reversal *ρ*(*i*,*i*) on *π* and let *π*^′^ denote the resulting permutation. We have that Vec(*π*^′^)=Vec(*π*) and $|V^{odd}_{\pi ^{\prime }}| = |V^{odd}_{\pi }|$. Moreover, since the sign of *π*_*i*_ is flipped without changing the parity of |*v*(*π*_*i*_)|, we have that $|V^{0^{-}}_{\pi ^{\prime }}| \geq |V^{0^{-}}_{\pi }| - 1 \geq |V^{0^{-}}_{\pi }| - 5$. Summing the previous equalities with this inequality we obtain (5).

Suppose now that we apply a signed 2-reversal *ρ*(*i*,*i*+1) on *π* and let *π*^′^ denote the resulting permutation. We have that Vec(*π*^′^)≥Vec(*π*)−2. Moreover, we have that $|V^{odd}_{\pi ^{\prime }}| \geq |V^{odd}_{\pi }| - 2$ and $|V^{0^{-}}_{\pi ^{\prime }}| \geq |V^{0^{-}}_{\pi }| - 2$, but since $V^{odd}_{\pi } \cap V^{0^{-}}_{\pi } = \emptyset $, we conclude that $|V^{odd}_{\pi ^{\prime }}| + |V^{0^{-}}_{\pi ^{\prime }}| \geq |V^{odd}_{\pi }| + |V^{0^{-}}_{\pi }| - 2 \geq |V^{odd}_{\pi }| + |V^{0^{-}}_{\pi }| - 3$. Summing the previous inequalities we obtain (5).

Finally, suppose that we apply a signed 3-reversal *ρ*(*i*,*i*+2) on *π* and let *π*^′^ denote the resulting permutation. We have that the parities of the lengths of the vectors do not change and hence $|V^{odd}_{\pi ^{\prime }}| = |V^{odd}_{\pi }|$. Moreover, we have that Vec(*π*^′^)≥Vec(*π*)−4 and $|V^{0^{-}}_{\pi ^{\prime }}| \geq |V^{0^{-}}_{\pi }| - 3$. It should be noted, however, that if *v*(*π*_*i*_) (or *v*(*π*_*i*+2_)) belongs to $V^{0^{-}}_{\pi }$, then Vec(*π*^′^)≥Vec(*π*)−2 because the length of *v*(*π*_*i*_)(or *v*(*π*_*i*+2_)) increases by 2 units. On the other hand, if neither *v*(*π*_*i*_) nor *v*(*π*_*i*+2_) belongs to $V^{0^{-}}_{\pi }$, then $|V^{0^{-}}_{\pi ^{\prime }}| \geq |V^{0^{-}}_{\pi }| - 1$. Therefore $\text {Vec}(\pi ^{\prime }) + |V^{0^{-}}_{\pi ^{\prime }}| \geq \text {Vec}(\pi) + |V^{0^{-}}_{\pi }| - 5$. Summing the previous equality with this inequality we obtain (5) and the lemma follows.

#### **Theorem2**.

The problem of sorting by short signed reversals is 5-approximable.

#### *Proof*.

Immediate from Lemmas 11 and 12.

Heath and Vergara [18] have described an algorithm for finding vector-opposite elements which runs in linear time on *n*, the size of the input permutation. Basically, what their algorithm does is to find vector-opposite elements *π*_*i*_ and *π*_*j*_ such that *v*(*π*_*i*_) is the rightmost right vector of *π*. Algorithm 1 is an adaptation of that algorithm. The difference between the two algorithms is that, given a signed permutation *π*≠*ι*_*n*_, Algorithm 1 guarantees that, if it returns a pair (*π*_*i*_,*π*_*i*+1_), then *π*_*i*_ and *π*_*i*+2_ are not 2-vector-opposite. Note that Algorithm 1 also runs in linear time on *n*.



Algorithm 2 sorts a signed permutation in two steps. While the signed permutation has vector-opposite elements, the algorithm finds a pair of them using Algorithm 1 and then switches their positions applying the signed short reversals described in Lemma 10. When the signed permutation has no vector-opposite elements, the algorithm applies signed 1-reversals until the signed permutation has no negative elements.



It follows from Theorem 2 that Algorithm 2 is a 5-approximation algorithm for the problem of sorting by short signed reversals. Regarding its time complexity, it suffices to compute the total cost of calls to lines 3, 4, and 7. The total cost of calls in line 3 equals the total cost for all calls to Algorithm 1. Although it runs in *O*(*n*) time and there are *O*(*n*^2^) vector-opposite elements in a signed permutation, we can provide the Algorithm 1 with enough information so that the costs of calls to this algorithm can be significantly reduced. Note that Algorithm 1 performs two scans in the signed permutation, one for each vector of the vector-opposite elements returned. By observing that a rightmost right vector remains a rightmost vector until it becomes a zero vector, it need not be searched again if the vector has not been zeroed. Thus, the scan for the rightmost vector needs to be performed only *O*(*n*) times. In addition, the total cost of scans for the left vector for the same right vector is bounded by the length of the right vector, also *O*(*n*). The total cost for all calls to Algorithm 1 with this refinement is thus *O*(*n*^2^). Each call to line 4 takes *O*(*m*) time, where *m*=*j*−*i*, and causes a strict decrease in Vec(*π*) of 2*m* units. Thus, the cost in this case is bounded by Vec(*π*) rather than the number of iterations performed in the while loop. As each vector has length at most *n*, we have that Vec(*π*)≤*n*^2^, meaning a cost of *O*(*n*^2^) time for the calls to line 4. Finally, we have that line 3 runs in *O*(*n*) time, therefore Algorithm 2 runs in *O*(*n*^2^) time.

We finish by noting that there exists a large class of signed permutations for which the approximation ratio of Algorithm 2 is much lower than its worst-case approximation ratio (Lemma 13). Moreover, based on the fact that the expected value of Vec(*π*) of a random signed permutation $\pi \in S^{\pm }_{n}$ is $\frac {n^{2} - 1}{3}$ (Lemma 15), we can conclude that the expected approximation ratio of Algorithm 2 for sorting a random signed permutation is also lower than the worst-case approximation ratio (Theorem 3). Just to make things clear, we define a random signed permutation as a random ordering of the elements {1,2,…,*n*}, with the added characteristic that the sign, + or −, of each element is also randomly chosen.

#### **Lemma13**.

Let *A*_2_(*π*) be the number of signed short reversals applied by Algorithm 2 for sorting a signed permutation $\pi \in S^{\pm }_{n}$. We have that $\frac {A_{2}(\pi)}{d_{\textit {ssr}}(\pi)} \leq 3$ when Vec(*π*)=0 or Vec(*π*)≥4*n*.

#### *Proof*.

We have two cases to consider:

- Vec(*π*)=0. In this case, we have that Algorithm 2 sorts *π* with $|V^{0^{-}}_{\pi }|$ signed 1-reversals. On the other hand, we have that $d_{\textit {ssr}}(\pi) \geq \frac {|V^{0^{-}}_{\pi }|}{3}$ because a signed short reversal cannot affect more than 3 elements at once. Therefore $\frac {A_{2}(\pi)}{d_{\textit {ssr}}(\pi)} \leq 3$.
- Vec(*π*)≥4*n*. In this case, we have seen that Algorithm 2 sorts *π* in two steps. First it applies signed 2-reversals and signed 3-reversals on *π* until Vec(*π*)=0 and then it applies signed 1-reversals on *π* until $V^{0^{-}}_{\pi } = 0$. Note that, in the first step, each signed short reversal applied by Algorithm 2 results in an average decrease in Vec(*π*) of at least 2 units. Hence Algorithm 2 applies at most $\frac {\text {Vec}(\pi)}{2}$ signed short reversals in the first step. Moreover, Algorithm 2 applies at most n signed 1-reversals in the second step because $|V^{0^{-}}_{\pi }| \leq n$. On the other hand, we have that $d_{\textit {ssr}}(\pi) \geq \frac {\text {Vec}(\pi)}{4}$ (Lemma 5). This analysis lead us to conclude that $\frac {A_{2}(\pi)}{d_{\textit {ssr}}(\pi)} \leq 2 + \frac {4n}{\text {Vec}(\pi)}$. Therefore $\frac {A_{2}(\pi)}{d_{\textit {ssr}}(\pi)} \leq 3$.

Since $\frac {A_{2}(\pi)}{d_{\textit {ssr}}(\pi)} \leq 3$ in both cases, the lemma follows.

In what follows, let Pr(|*v*(*π*_*i*_)|=*j*) denote the probability that |*v*(*π*_*i*_)| is equal to *j* and $\mathbb {E}(X)$ denote the expected value of a random variable *X*.

#### **Lemma14**.

Let $\pi \in S^{\pm }_{n}$ be a random signed permutation. Then $\sum _{i=1}^{n} \Pr (\lvert \,\!v(\pi _{i})\rvert = j) = \frac {2(n-j)}{n}$ for 1≤*j*≤*n*−1.

#### *Proof*.

We have that $\lvert S^{\pm }_{n} \rvert = n!2^{n}$ and for each 1≤*k*≤*n*, there are (*n*−1)!2^*n*^ signed permutations for which |*π*_*i*_|=*k*. Then

$${}\Pr(\lvert\,\!v(\pi_{i})\rvert \,=\, j) \,=\, \left\{ \begin{array}{ll} \frac{1}{n} & \quad \text{if}~~ j = 0,\\ \frac{2}{n} & \quad \text{if}~~ i+j \leq n ~~\text{and}~~ i-j \geq 1,\\ \frac{1}{n} & \quad \text{if}~~ i+j > n ~~\text{or}~~ i-j < 1 ~~\text{but not both,}\\ 0 & \quad \text{otherwise,} \end{array} \right. $$

for 0 ≤*j*≤*n*−1. In order to evaluate $\sum _{i=1}^{n} \Pr (\lvert \,\!v(\pi _{i})\rvert = j)$ for a given *j*, we consider two cases:

- $1 \leq j < \frac {n}{2}$. In this case, we have that
  $$\Pr(\lvert\,\!v(\pi_{i})\rvert = j) = \left\{ \begin{array}{l l} \frac{1}{n} & \quad \text{if}~~ 1 \leq i \leq j,\\ \frac{1}{n} & \quad \text{if} ~~n~~ - j + 1 \leq i \leq n,\\ \frac{2}{n} & \quad \text{otherwise.} \end{array} \right. $$
  Therefore, we have that $\sum _{i=1}^{n} \Pr (\lvert \,\!v(\pi _{i})\rvert = j) = \frac {j}{n} + \frac {j}{n}$$ + \frac {2(n-2j)}{n} = \frac {2(n-j)}{n}$.
- $\frac {n}{2} \leq j \leq n$. In this case, we have that
  $$\Pr(\lvert\,\!v(\pi_{i})\rvert = j) = \left\{ \begin{array}{l l} \frac{1}{n} & \quad \text{if} ~~1 \leq i \leq n - j,\\ \frac{1}{n} & \quad \text{if}~~ j + 1 \leq i \leq n,\\ 0 & \quad \text{otherwise.} \end{array} \right. $$
  Therefore, we have that $\sum _{i=1}^{n} \Pr (\lvert \,\!v(\pi _{i})\rvert = j) = \frac {n-j}{n} + $$ \frac {n-j}{n} = \frac {2(n-j)}{n}$.

Since in both cases $\sum _{i=1}^{n} \Pr (\lvert \,\!v(\pi _{i})\rvert = j) = \frac {2(n-j)}{n}$ holds, the lemma follows.

#### **Lemma15**.

Let $\pi \in S^{\pm }_{n}$ be a random signed permutation. Then $\mathbb {E}(\text {Vec}(\pi)) = \frac {n^{2} - 1}{3}$.

#### *Proof*.

Given that $\mathbb {E}(\lvert v(\pi _{i})\rvert)=\sum _{j=0}^{n-1} j\Pr (\lvert v(\pi _{i}) \rvert = j)$, we have that

$$\begin{array}{rcl} \mathbb{E}(\text{Vec}(\pi)) & = & \mathbb{E}\left(\sum_{i=1}^{n} \lvert\,\!v(\pi_{i})\rvert\right)\\ & = & \sum_{i=1}^{n} \mathbb{E}(\lvert\,\!v(\pi_{i})\rvert) \\ [2pt] & = & \sum_{i=1}^{n}\sum_{j=0}^{n-1} j\Pr(\lvert\,\!v(\pi_{i})\rvert = j) \\ & = & \sum_{j=1}^{n-1}j\sum_{i=1}^{n} \Pr(\lvert\,\!v(\pi_{i})\rvert = j) \\ & = & \sum_{j=1}^{n-1}j\frac{2(n-j)}{n} \\ & = & 2\sum_{j=1}^{n-1}j - \frac{2}{n}\sum_{j=1}^{n-1}j^{2} \\ & = & 2\left(\frac{n^{2}-n}{2}\right) - \frac{2}{n}\left(\frac{(n-1)n(2n-1)}{6}\right) \\ & = & n^{2} - n - \frac{2n^{2} - 3n + 1}{3} \\ & = & \frac{n^{2} - 1}{3}, \end{array} $$

and the lemma follows.

#### **Theorem3**.

The expected approximation ratio of Algorithm 2 for sorting a random signed permutation $\pi \in S^{\pm }_{n}$ is no greater than 3 for *n*≥13.

#### *Proof*.

According to Lemma 13, we have that the approximation ratio of Algorithm 2 for sorting a given signed permutation $\sigma \in S^{\pm }_{n}$ is no greater than 3 when Vec(*σ*)≥4*n*. Since we know that the expected value of Vec(*π*) of a random signed permutation $\pi \in S^{\pm }_{n}$ is $\frac {n^{2} - 1}{3}$ (Lemma 15), we conclude that the expected approximation ratio of Algorithm 2 for sorting *π* is no greater than 3 if $\frac {n^{2} - 1}{3} \geq 4n$. This inequality holds when *n*≥13, and the theorem follows.

## Sorting by bounded operations

In this section, we present a polynomial-time solution for the problem of sorting by super short operations and a 3-approximation algorithm for the problem of sorting by short operations. Before we present the main results, we first introduce a useful tool for tackling these problems, the *permutation graph*. This tool was also used by Heath and Vergara [20] for dealing with the problem of sorting by short transpositions.

### The permutation graph

**Figure 2:**
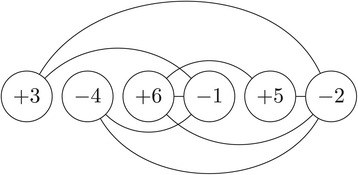
**Permutation graph.** Permutation graph of the signed permutation (+3−4+6−1+5−2).

The *permutation graph* of a permutation $\pi \in S^{\pm }_{n}$ is the undirected graph *G*_*π*_=(*V*,*E*), where *V*={*π*_1_,*π*_2_,…,*π*_*n*_} and *E*={(*π*_*i*_,*π*_*j*_):*i*<*j* and |*π*_*i*_|>|*π*_*j*_|}. In other words, *G*_*π*_ is an undirected graph whose vertex set is formed by the elements of *π* and edge set is formed by the inversions in *π*. Figure 2 illustrates *G*_*π*_ for *π*=(+3−4+6−1+5−2).

Given a signed permutation *π*, we denote the number of connected components (or simply components) of *G*_*π*_ by *c*(*π*). Moreover, we say that a component of *G*_*π*_ is *odd* if it contains an odd number of negative elements (vertices) and we say it is *even* otherwise. The number of odd components of *G*_*π*_ is denoted by *c*_*odd*_(*π*). Lastly, we say that an edge of *G*_*π*_ is a *cut-edge* if its deletion increases the number of components of *G*_*π*_.

### Sorting by signed super short operations

From the proof of Lemma 2, we have that a super short operation can eliminate at most one inversion of a signed permutation. This means that, for sorting a signed permutation *π*, we have to apply Inv(*π*) super short operations (*i.e.* 2-reversals and (1, 1)-transpositions) plus a given number of signed 1-reversals in order to flip the signs of the remaining negative elements. As before, the question is: how many signed 1-reversals do we have to apply? As Lemmas 16 and 17 show, the answer is *c*_*odd*_(*π*).

#### **Lemma16**.

Let $\pi \in S^{\pm }_{n}$ be a signed permutation. Then, we have that *d*_*ssso*_(*π*)≤Inv(*π*)+*c*_*odd*_(*π*).

#### *Proof*.

It suffices to prove that it is always possible to apply a signed super short operation on *π*≠*ι*_*n*_ in such a way that the resulting permutation *π*^′^ satisfies

(6) $$\begin{array}{@{}rcl@{}} \text{Inv}(\pi^{\prime}) + c_{odd}(\pi^{\prime}) \leq \text{Inv}(\pi) + c_{odd}(\pi) - 1. \end{array} $$

If Inv(*π*)=0, then each component of *G*_*π*_ is a single vertex. Therefore, we can sort *π* with *c*_*odd*_(*π*) signed 1-reversals and (6) holds.

If Inv(*π*)>0, then there exists an edge *e*=(*π*_*i*_,*π*_*i*+1_) in *G*_*π*_ (Lemma 1). Suppose first that e is not a cut-edge and that we apply the (1, 1)-transposition *ρ*(*i*,*i*+1,*i*+2) on *π*, obtaining the permutation *π*^′^. We have that Inv(*π*^′^)=Inv(*π*)−1. Moreover, since e is not a cut-edge, we have that the vertex sets of the components of $G_{\pi ^{\prime }}$ are the same as of the components of *G*_*π*_. This means that *c*_*odd*_(*π*^′^)=*c*_*odd*_(*π*). Summing both equalities we obtain (6).

Now, suppose that *e* is a cut-edge and let *C* denote the component of *G*_*π*_ which contains *e*. Moreover, let *C*_1_ and *C*_2_ denote the components of *C*−*e* and assume, without loss of generality, that *π*_*i*_∈*C*_1_. We have three cases to consider:

- *C*_1_ and *C*_2_ are both even. Note that *C* is even. Apply the (1, 1)-transposition *ρ*(*i*,*i*+1,*i*+2) on *π* and let *π*^′^ denote the resulting permutation. Then, we have that Inv(*π*^′^)=Inv(*π*)−1 and that *c*_*odd*_(*π*^′^)=*c*_*odd*_(*π*). Summing both equalities we obtain (6).
- *C*_1_ and *C*_2_ have distinct parities. Note that *C* is odd. Apply the (1, 1)-transposition *ρ*(*i*,*i*+1,*i*+2) on *π* and let *π*^′^ denote the resulting permutation. Then, we have that Inv(*π*^′^)=Inv(*π*)−1 and that *c*_*odd*_(*π*^′^)=*c*_*odd*_(*π*). Summing both equalities we obtain (6).
- *C*_1_ and *C*_2_ are both odd. Note that *C* is even. Apply the signed 2-reversal *ρ*(*i*,*i*+1) on *π* and let *π*^′^ denote the resulting permutation. Then, we have that Inv(*π*^′^)=Inv(*π*)−1. Moreover, we have that *c*_*odd*_(*π*^′^)=*c*_*odd*_(*π*) because *C*_1_ and *C*_2_ become even after the signed reversal is applied on *π*. Summing both equalities we obtain (6).

Since it is always possible to apply a signed super short operation on *π* in such a way that the resulting permutation *π*^′^ satisfies (6), the lemma follows.

#### **Lemma17**.

Let $\pi \in S^{\pm }_{n}$ be a signed permutation. Then *d*_*ssso*_(*π*)≥Inv(*π*)+*c*_*odd*_(*π*).

#### *Proof*.

It suffices to prove that if we apply an arbitrary super short operation on *π*, then the resulting permutation *π*^′^ satisfies

(7) $$\begin{array}{@{}rcl@{}} \text{Inv}(\pi^{\prime}) + c_{odd}(\pi^{\prime}) \geq \text{Inv}(\pi) + c_{odd}(\pi) - 1. \end{array} $$

Suppose first that we apply a signed 1-reversal *ρ*(*i*,*i*) and let *π*^′^ denote the resulting permutation. Then, we have that Inv(*π*^′^)=Inv(*π*). Moreover, since the component containing *π*_*i*_ may become even, we have that *c*_*odd*_(*π*^′^)≥*c*_*odd*_(*π*)−1. Summing the previous equality with this inequality we obtain (7).

Now, suppose that we apply the (1, 1)-transposition *ρ*(*i*,*i*+1,*i*+2) on *π* and let *π*^′^ denote the resulting permutation. We have two cases to consider:

- (*π*_*i*_,*π*_*i*+1_) is not an inversion. In this case, we have that Inv(*π*^′^)=Inv(*π*)+1. On the other hand, by adding a new edge, we may eliminate two odd components, therefore *c*_*odd*_(*π*^′^)≥*c*_*odd*_(*π*)−2. Summing the previous equality with this inequality we obtain (7).
- (*π*_*i*_,*π*_*i*+1_) is an inversion. In this case, we have that Inv(*π*^′^)=Inv(*π*)−1. Moreover, let *e*=(*π*_*i*_,*π*_*i*+1_) be an edge of *G*_*π*_ and let *C* be the component of *G*_*π*_ containing *e* and. We further divide our analysis into two subcases:
  i) *e* is not a cut-edge. In this case, we have that *c*_*odd*_(*π*^′^)=*c*_*odd*_(*π*) because the parity of the component *C*−*e* is the same as of *C*, therefore (7) holds.
  ii) *e* is a cut-edge. In this case, let *C*_1_ and *C*_2_ denote the components of *C*−*e*. If *C* is odd, then either *C*_1_ or *C*_2_ is odd. If *C* is even, then either *C*_1_ and *C*_2_ are both odd or *C*_1_ and *C*_2_ are both even. In any case, we have that *c*_*odd*_(*π*^′^)≥*c*_*odd*_(*π*), therefore (7) holds.

Finally, suppose that we apply the signed 2-reversal *ρ*(*i*,*i*+1) on *π* and let *π*^′^ denote the resulting permutation. By making use of an argument analogous to the one in the previous paragraph, we conclude that *π*^′^ satisfies (7) and the lemma follows.

#### **Theorem4**.

Let $\pi \in S^{\pm }_{n}$ be a signed permutation. Then, *d*_*ssso*_(*π*)=Inv(*π*)+*c*_*odd*_(*π*).

#### *Proof*.

Immediate from Lemmas 16 and 17.

Let *π* be a signed permutation. From the proof of Lemma 17, we can conclude that a super short operation cannot decrease the value of *c*_*odd*_(*π*) if it is applied on an inversion in *π*. Moreover, from the proof of Lemma 16, we can conclude that if a (1, 1)-transposition increases the value of *c*_*odd*_(*π*) when applied on an inversion in *π*, then it is possible to apply a signed 2-reversal on this inversion in such a way that *c*_*odd*_(*π*) remains unaltered. These observations lead us to the following optimal algorithm for sorting by signed super short operations (Algorithm 3).



The time complexity of Algorithm 3 depends on the time complexity of the algorithm used to compute the value of *c*_*odd*_(*π*). A straightforward algorithm is to traverse *G*_*π*_ with a depth-first search and count the number of odd components. Such an algorithm runs in *O*(*n*^2^) time. It is possible, however, to count the number of odd components in *G*_*π*_ in *O*(*n*) time.

Koh and Ree [30] have studied the permutation graph of unsigned permutations and have demonstrated some useful properties about them. Since the permutation graph of the signed permutation $\pi \in S^{\pm }_{n}$ is isomorphic to the permutation graph of the unsigned permutation (|*π*_1_||*π*_2_|…|*π*_*n*_|), we are able to translate those properties to the permutation graph of signed permutations. In particular, Lemma 18 represents the translation of one of those properties.

#### **Lemma18**.

Let $\pi \in S^{\pm }_{n}$ be a signed permutation. The vertex sets of the components of *G*_*π*_ are of the form *C*_1_={*π*_1_,*π*_2_,…,*π*_*k*_},*C*_2_={*π*_*k*+1_,*π*_*k*+2_,…,*π*_*l*_},…,*C*_*t*_={*π*_*m*+1_,*π*_*m*+2_,…,*π*_*n*_}. Moreover, we have that {|*π*_1_|,|*π*_2_|,…,|*π*_*k*_|}={1,2,…,*k*},{|*π*_*k*+1_|,|*π*_*k*+2_|,…,|*π*_*l*_|}={*k*+1,*k*+2,…,*l*},…,{|*π*_*m*+1_|,|*π*_*m*+2_|,…,|*π*_*n*_|}={*m*+1,*m*+2,…,*n*}.

We say that a contiguous sequence of elements *π*_*i*_*π*_*i*+1_…*π*_*j*_,*i*≤*j*, of a signed permutation *π* is a *complete substring* if {|*π*_*i*_|,|*π*_*i*+1_|,…,|*π*_*j*_|}={*i*,*i*+1,…,*j*}. From Lemma 18, we have that the vertex set of a component of *G*_*π*_ forms a complete substring. Furthermore, assume that {*π*_*i*_,*π*_*i*+1_,…,*π*_*j*_} is the vertex set of a component of *G*_*π*_. We claim that *π*_*i*_*π*_*i*+1_…*π*_*j*_ is the minimum complete substring that starts with *π*_*i*_. For the sake of contradiction, suppose that there exists a complete substring *π*_*i*_*π*_*i*+1_…*π*_*k*_ such that *k*<*j*. We have that *π*_*l*_>*π*_*m*_ for every *i*≤*l*≤*k* and *k*+1≤*m*≤*j*. Therefore there does not exist any edge in *G*_*π*_ connecting the elements in {*π*_*i*_,*π*_*i*+1_,…,*π*_*k*_} with the elements in {*π*_*k*+1_,*π*_*k*+2_,…,*π*_*j*_}. But this contradicts our hypothesis that {*π*_*i*_,*π*_*i*+1_,…,*π*_*j*_} is the vertex set of a component of *G*_*π*_.

From the discussion of the last paragraph, we can design the following algorithm for finding the vertex sets of the components of the permutation graph of a signed permutation $\pi \in S^{\pm }_{n}$. Find the minimum complete substring *π*_1_*π*_2_…*π*_*k*_ starting with *π*_1_ and let *C*_1_={*π*_1_,*π*_2_,…,*π*_*k*_} be a component of *G*_*π*_. If *k*<*n*, then find the minimum complete substring *π*_*k*+1_*π*_*k*+2_…*π*_*l*_ starting with *π*_*k*+1_ and let *C*_2_={*π*_*k*+1_,*π*_*k*+2_,…,*π*_*l*_} be another component of *G*_*π*_. Continue with this process until all elements have been assigned to a component. It remains to show how to find the minimum complete substring *π*_*i*_*π*_*i*+1_…*π*_*j*_ starting with *π*_*i*_. Note that i is the least element and j is the largest element of the set *S*={|*π*_*i*_|,|*π*_*i*+1_|,…,|*π*_*j*_|}. Since all integers in the interval [*i*,*j*] are in *S*, we have that |*S*|=*j*−*i*+1. This fact give us the necessary and sufficient condition for knowing when we have found the last element of the minimum complete substring starting with *π*_*i*_. The complete algorithm is detailed below (Algorithm 4).



Algorithm 4 performs a linear scan on the positions of the permutation $\pi \in S^{\pm }_{n}$, and so it runs in *O*(*n*). With the vertex sets of the components of *G*_*π*_, it is easy to count the number of odd components in *G*_*π*_ in *O*(*n*) time. Returning to Algorithm 3, we can see that lines 4-9 run in *O*(*n*) time. Since the while loop iterates a total of *O*(*n*^2^) times and line 12 runs in *O*(*n*) time, we can conclude that Algorithm 3 runs in *O*(*n*^3^) time. We remark that the value of *d*_*ssso*_(*π*) can be computed in $O(n\,\sqrt {\log {n}})$ time because computing *c*_*odd*_(*π*) takes *O*(*n*) time and computing Inv(*π*) takes $O(n\,\sqrt {\log {n}})$ time [29].

### Sorting by signed short operations

A trivial algorithm for the problem of sorting by signed short operations is the optimal algorithm for the problem of sorting by signed super short operations. From the lower bound of Lemma 19, it follows that this algorithm is a 4-approximation algorithm. In addition, we have that this approximation bound is tight. For instance, we need 4 signed super short operations for sorting the signed permutation (−3−2−1), but one signed 3-reversal is sufficient for sorting it.

#### **Lemma19**.

Let $\pi \in S^{\pm }_{n}$ be a signed permutation. Then, $d_{\textit {sso}}(\pi) \geq \frac {\text {Inv}(\pi) + c_{\textit {odd}}(\pi)}{4}$.

#### *Proof*.

It suffices to prove that if we apply an arbitrary short operation on *π*, then the resulting permutation *π*^′^ satisfies

(8) $$\begin{array}{@{}rcl@{}} \text{Inv}(\pi^{\prime}) + c_{odd}(\pi^{\prime}) \geq \text{Inv}(\pi) + c_{odd}(\pi) - 4. \end{array} $$

From the proof of Lemma 17, we have that (8) holds in case we apply a super short operation on *π*. So, suppose that we apply a short operation *ρ* on *π* which acts on the elements *π*_*i*_,*π*_*i*+1_, and *π*_*i*+2_. Moreover, let *π*^′^ denote the resulting permutation. We have three cases to consider:

- *π*_*i*_,*π*_*i*+1_, and *π*_*i*+2_ belong to the same component. In this case, we have that Inv(*π*^′^)≥Inv(*π*)−3 and *c*_*odd*_(*π*^′^)≥*c*_*odd*_(*π*)−1, therefore (8) holds.
- two elements in {*π*_*i*_,*π*_*i*+1_,*π*_*i*+2_} belong to a component *C*_1_ and the remaining element belongs to a component *C*_2_. In this case, we have that Inv(*π*^′^)≥Inv(*π*)−1 and *c*_*odd*_(*π*^′^)≥*c*_*odd*_(*π*)−2, therefore (8) holds.
- *π*_*i*_,*π*_*i*+1_, and *π*_*i*+2_ belong to distinct components. In this case, we have that Inv(*π*^′^)=Inv(*π*)+3 and *c*_*odd*_(*π*^′^)≥*c*_*odd*_(*π*^′^)−3, therefore (8) holds.

Since (8) holds in any case, the lemma follows.

Given a signed permutation *π*, let $c^{t}_{\textit {odd}}(\pi)$ be the number of odd components of *G*_*π*_ which have exactly *t* vertices. By just considering the odd components having at most two vertices, we can obtain better bounds on the signed short operation distance of a signed permutation *π* (Lemmas 21 and 22). These bounds lead to a 3-approximation for the problem of sorting by signed short reversals (Theorem 5). We note that the upper bound given in Lemma 21 relies on the fact that we can establish an isomorphism between a component with *m* vertices and the permutation graph of a signed permutation $\sigma \in S^{\pm }_{m}$ (Lemma 20).

#### **Lemma20**.

Let $\pi \in S^{\pm }_{n}$ be a signed permutation and let *C*=(*V*_*C*_,*E*_*C*_) be a component of *G*_*π*_ with *m* vertices. Then, there exists a signed permutation $\sigma \in S^{\pm }_{m}$ such that *G*_*σ*_ is isomorphic to *C*.

#### *Proof*.

By Lemma 18, we have that if *V*_*C*_={*π*_*i*+1_,*π*_*i*+2_,…,*π*_*i*+*m*_}, then {|*π*_*i*+1_|,|*π*_*i*+2_|,…,|*π*_*i*+*m*_|}= {*i*+1,*i*+2,…,*i*+*m*}. Let $\sigma \in S^{\pm }_{m}$ be a signed permutation such that

$$\sigma_{j} = \left\{ \begin{array}{l l} \pi_{i+j} - i & \quad \text{if} ~~\pi_{i+j} > 0\\ \pi_{i+j} + i & \quad \text{if} ~~\pi_{i+j} < 0 \end{array} \right. $$

for all *j*∈{1,2,…,*m*}. We claim that the bijective function *f*(*π*_*i*+*x*_)=*σ*_*x*_ is an isomorphism between *C* and *G*_*σ*_. To see this, firstly note that *π*_*i*+*x*_ is a negative vertex if, and only if, *σ*_*x*_ is a negative vertex. Secondly, let *k* and *l* be to integers such that 1 ≤*k*<*l*≤*m*. Note that (*π*_*i*+*k*_,*π*_*i*+*l*_) is an edge of *C* if, and only if, (*σ*_*k*_,*σ*_*l*_) is an edge of *G*_*σ*_, and so the lemma follows.

#### **Lemma21**.

Let $\pi \in S^{\pm }_{n}$ be a signed permutation. Then $d_{\textit {sso}}(\pi) \leq \text {Inv}(\pi) + c^{2}_{\textit {odd}}(\pi) + c^{1}_{\textit {odd}}(\pi)$.

#### *Proof*.

It suffices to prove that it is always possible to apply a sequence of *t*>0 signed short operations on *π*≠*ι*_*n*_ in such a way that the resulting permutation *π*^′^ satisfies

(9) $$ {\small{\begin{aligned} {} \text{Inv}(\pi^{\prime}) \,+\, c^{2}_{odd}(\pi^{\prime}) + c^{1}_{odd}(\pi^{\prime}) \!\leq\! \text{Inv}(\pi) \,+\, c^{2}_{odd}(\pi) \,+\, c^{1}_{odd}(\pi) - t. \end{aligned}}}\vspace*{-8pt}  $$

If Inv(*π*) = 0, then each component of *G*_*π*_ is a single vertex. Therefore, we can apply $c^{1}_{\textit {odd}}$(*π*) signed 1-reversals and (9) holds.

If Inv(*π*)>0, then there exists an edge *e*=(*π*_*i*_,*π*_*i*+1_) in *G*_*π*_ (Lemma 1). Let *C* denote the component of *G*_*π*_ which contains *e* and assume that *C* contains *m* vertices. We have four cases to consider:

- *m*≥5. In this case, we further divide our analysis into two subcases:
  i) *e* is not a cut-edge. In this case, apply the (1, 1)-transposition *ρ*(*i*,*i*+1,*i*+2) on *π* and let *π*^′^ denote the resulting permutation. Then, we have that $\text {Inv}(\pi ^{\prime }) = \text {Inv} (\pi) - 1, c^{2}_{\textit {odd}}(\pi ^{\prime }) = c^{2}_{\textit {odd}}(\pi)$, and $c^{1}_{\textit {odd}}(\pi ^{\prime }) = c^{1}_{\textit {odd}}(\pi)$. Therefore (9) holds.
  ii) *e* is a cut-edge. In this case, let *C*_1_ and *C*_2_ denote the components of *C*−*e*. Moreover, let *m*_1_ be the number of vertices in *C*_1_ and let *m*_2_ be the number of vertices in *C*_2_. If *m*_1_≥ 3 and *m*_2_≥ 3, then apply the (1, 1)-transposition *ρ*(*i*,*i*+1,*i*+2) on *π* and let *π*^′^ denote the resulting permutation. We have that $\text {Inv}(\pi ^{\prime }) = \text {Inv}(\pi) - 1, c^{2}_{\textit {odd}}(\pi ^{\prime }) = c^{2}_{\textit {odd}}(\pi)$, and $c^{1}_{\textit {odd}}(\pi ^{\prime }) = c^{1}_{\textit {odd}}(\pi)$. So, without loss of generality, assume that *m*_1_≤2. Note that *m*_2_≥3 because *m*_1_+*m*_2_=*m*≥5. If *C*_1_ is even, then apply the (1, 1)-transposition *ρ*(*i*,*i*+1,*i*+2) on *π* and let *π*^′^ denote the resulting permutation. We have that $\text {Inv}(\pi ^{\prime }) = \text {Inv}(\pi) - 1, c^{2}_{\textit {odd}}(\pi ^{\prime }) = c^{2}_{\textit {odd}}(\pi)$, and $c^{1}_{\textit {odd}}(\pi ^{\prime }) = c^{1}_{\textit {odd}}(\pi)$. Otherwise, if *C*_1_ is odd, apply the signed the 2-reversal *ρ*(*i*,*i*+1) on *π* and let *π*^′^ denote the resulting permutation. We have that $\text {Inv}(\pi ^{\prime }) = \text {Inv}(\pi) - 1, c^{2}_{\textit {odd}}(\pi ^{\prime }) = c^{2}_{\textit {odd}}(\pi)$, and $c^{1}_{\textit {odd}}(\pi ^{\prime }) = c^{1}_{\textit {odd}}(\pi)$. In any case, we have that the resulting permutation *π*^′^ satisfies (9).
- *m*= 4. According to Lemma 20, there exists a signed permutation $\sigma \in S^{\pm }_{4}$ such that *G*_*σ*_ is isomorphic to *C*. We have verified that every permutation $\sigma \in S^{\pm }_{4}$ for which *c*(*σ*)= 1 can be sorted with at most Inv(*σ*) signed short operations, therefore it is possible to apply a sequence of signed short operations on *C* in such a way that the resulting permutation *π*^′^ satisfies (9).
- *m*= 3. Analogous to case b).
- *m*= 2. In this case, we further divide our analysis into three subcases:
  i) *π*_*i*_ and *π*_*i*+1_ are both negatives. In this case, apply the signed the 2-reversal *ρ*(*i*,*i*+1) on *π* and let *π*^′^ denote the resulting permutation. We have that $\text {Inv}(\pi ^{\prime }) = \text {Inv}(\pi) - 1, c^{2}_{\textit {odd}}(\pi ^{\prime }) = c^{2}_{\textit {odd}}(\pi)$, and $c^{1}_{\textit {odd}}(\pi ^{\prime }) = c^{1}_{\textit {odd}}(\pi)$, therefore (9) holds.
  ii) *π*_*i*_ and *π*_*i*+1_ have distinct signs. In this case, apply the (1, 1)-transposition *ρ*(*i*,*i*+1,*i*+2) on *π* and let *π*^′^ denote the resulting permutation. Then, we have that $\text {Inv}(\pi ^{\prime }) = \text {Inv}(\pi) - 1, c^{2}_{\textit {odd}}(\pi ^{\prime }) = c^{2}_{\textit {odd}}(\pi) - 1$, and $c^{1}_{\textit {odd}}(\pi ^{\prime }) = c^{1}_{\textit {odd}}(\pi) + 1$, therefore (9) holds.
  iii) *π*_*i*_ and *π*_*i*+1_ are both positives. In this case, apply the (1, 1)-transposition *ρ*(*i*,*i*+1,*i*+2) on *π* and let *π*^′^ denote the resulting permutation. Then, we have that $\text {Inv}(\pi ^{\prime }) = \text {Inv}(\pi) - 1, c^{2}_{\textit {odd}}(\pi ^{\prime }) = c^{2}_{\textit {odd}}(\pi)$, and $c^{1}_{\textit {odd}}(\pi ^{\prime }) = c^{1}_{\textit {odd}}(\pi)$, therefore (9) holds.

Since it is always possible to apply a sequence of signed short operations on *π* in such a way that the resulting permutation *π*^′^ satisfies (9), the lemma follows.

#### **Lemma22**.

Let $\pi \in S^{\pm }_{n}$ be a signed permutation. Then, we have that $d_{\textit {sso}}(\pi) \geq \frac {\text {Inv}(\pi) + c^{2}_{\textit {odd}}(\pi) + c^{1}_{\textit {odd}}(\pi)}{3}$.

#### *Proof*.

It suffices to prove that if we apply an arbitrary short operation on *π*, then the resulting permutation *π*^′^ satisfies

(10) $$ {\small{\begin{aligned} {} \text{Inv}(\pi^{\prime}) + c^{2}_{odd}(\pi^{\prime}) + c^{1}_{odd}(\pi^{\prime}) \!\geq\! \text{Inv}(\pi) \!+ c^{2}_{odd}(\pi) \,+\, c^{1}_{odd}(\pi) \,-\, 3. \end{aligned}}}  $$

Suppose first that we apply a signed 1-reversal *ρ*(*i*,*i*) and let *π*^′^ denote the resulting permutation. Then, we have that Inv(*π*^′^)=Inv(*π*). Moreover, since *π*_*i*_ can belong to an odd component with at most two vertices, we have that $c^{2}_{\textit {odd}}(\pi ^{\prime })+ c^{1}_{\textit {odd}}(\pi ^{\prime }) \geq c^{2}_{\textit {odd}}(\pi) + c^{1}_{\textit {odd}}(\pi) - 1$, therefore (10) holds.

Now, suppose that we apply a super short operation *ρ* on *π* which acts on the elements *π*_*i*_ and *π*_*i*+1_, and let *π*^′^ denote the resulting permutation. We have two cases to consider:

- *π*_*i*_ and *π*_*i*+1_ belong to the same component. In this case, we have that Inv(*π*^′^)=Inv(*π*)− 1 and $c^{2}_{\textit {odd}}(\pi ^{\prime }) + c^{1}_{\textit {odd}}(\pi ^{\prime }) \geq c^{2}_{\textit {odd}}(\pi) + c^{1}_{\textit {odd}}(\pi)$, and (10) holds.
- *π*_*i*_ and *π*_*i*+1_ belong to distinct components. In this case, we have that Inv(*π*^′^)=Inv(*π*)+1 and $c^{2}_{\textit {odd}}(\pi ^{\prime }) + c^{1}_{\textit {odd}}(\pi ^{\prime }) \geq c^{2}_{\textit {odd}}(\pi) + c^{1}_{\textit {odd}}(\pi) - 2$. Therefore (10) holds.

Finally, suppose that we apply a short operation *ρ* on *π* which acts on the elements *π*_*i*_, *π*_*i*+1_, and *π*_*i*+2_. Moreover, let *π*^′^ denote the resulting permutation. We have three cases to consider:

- *π*_*i*_,*π*_*i*+1_, and *π*_*i*+2_ belong to the same component. In this case, we have that Inv(*π*^′^)≥Inv(*π*)−3 and $c^{2}_{\textit {odd}}(\pi ^{\prime }) + c^{1}_{\textit {odd}}(\pi ^{\prime }) \geq c^{2}_{\textit {odd}}(\pi) + c^{1}_{\textit {odd}}(\pi)$. Therefore (10) holds.
- two elements in {*π*_*i*_,*π*_*i*+1_,*π*_*i*+2_} belong to the component *C*_1_ and the remaining element belongs to the component *C*_2_. In this case, we have that Inv(*π*^′^)≥Inv(*π*)−1 and $c^{2}_{\textit {odd}}(\pi ^{\prime }) + c^{1}_{\textit {odd}}(\pi ^{\prime }) \geq c^{2}_{\textit {odd}}(\pi) + c^{1}_{\textit {odd}}(\pi) - 2$, and (10) holds.
- *π*_*i*_,*π*_*i*+1_, and *π*_*i*+2_ belong to distinct components. In this case, we have that *π*_*i*_<*π*_*i*+1_<*π*_*i*+2_, thus Inv(*π*^′^)=Inv(*π*)+3. Moreover, we have that $c^{2}_{\textit {odd}}(\pi ^{\prime }) + c^{1}_{\textit {odd}}(\pi ^{\prime }) \geq c^{2}_{\textit {odd}}(\pi) + c^{1}_{\textit {odd}}(\pi) - 3$. Therefore (10) holds.

Since (10) holds in every case, the lemma follows.

#### **Theorem5**.

The problem of sorting by short signed operations is 3-approximable.

#### *Proof*.

Immediate from Lemmas 21 and 22.

Let *π* be a signed permutation. From the proof of Lemma 21, we can conclude that as long as Inv(*π*)> 0, we can apply a sequence of short operations that eliminates inversions and keeps the value of $c^{2}_{\textit {odd}}(\pi) + c^{1}_{\textit {odd}}(\pi)$ unchanged. When Inv(*π*)= 0, we can sort *π* applying $c^{1}_{\textit {odd}}(\pi)$ signed 1-reversals. This is precisely what Algorithm 5 does.



It follows from Theorem 5 that Algorithm 5 is a 3-approximation algorithm for the problem of sorting by short reversals. Regarding its time complexity, we have that each iteration of the while loop takes *O*(*n*) time. Since the while loop iterates a total of *O*(*n*^2^) times and line 26 runs in *O*(*n*) time, we can conclude that Algorithm 5 runs in *O*(*n*^3^) time.

## Experimental results

We have implemented Algorithms 2 and 5, and we have audited them using GRAAu [31]. The audit consists of comparing the distance computed by an algorithm with the rearrangement distance for every $\pi \in S^{\pm }_{n}$, 1 ≤*n*≤ 10. The results are presented in Tables 1 and 2, where *n* is the size of the permutations, *Avg. Ratio* is the average of the ratios between the distance returned by an algorithm and the rearrangement distance, *Max. Ratio* is the greatest ratio among all the ratios between the distance returned by an algorithm and the rearrangement distance, and *Exact* is the percentage of distances returned by the algorithm that is exactly the rearrangement distance.

**Table 1:** **Results obtained from the audit of the implementation of Algorithm 2**

**n**	**Avg. ratio**	**Max. ratio**	**Exact**
1	1.00	1.00	100.00%
2	1.00	1.00	100.00%
3	1.13	2.50	77.08%
4	1.18	3.00	60.16%
5	1.24	3.00	41.04%
6	1.28	3.00	26.04%
7	1.31	3.00	15.06%
8	1.34	3.00	8.00%
9	1.35	3.00	3.93%
10	1.37	3.00	1.79%

**Table 2:** **Results obtained from the audit of the implementation of Algorithm 5**

**n**	**Avg. ratio**	**Max. ratio**	**Exact**
1	1.00	1.00	100.00%
2	1.00	1.00	100.00%
3	1.04	1.50	91.67%
4	1.02	1.50	93.75%
5	1.31	3.00	46.41%
6	1.54	3.00	19.11%
7	1.73	3.00	7.13%
8	1.87	3.00	2.50%
9	1.99	3.00	0.75%
10	2.08	3.00	0.20%

Besides providing the *Max. Ratio*, GRAAu also provides up to 50 permutations for which the algorithms achieved this ratio. These permutations can be used to obtain lower bounds on the theoretical approximation ratios of Algorithms 2 and 5. This is precisely what Lemmas 23 and 24 do. Observe that, in the case of Algorithm 5, the lower bound matches the upper bound, so we can conclude that its approximation ratio is tight (Lemma 25).

### **Lemma23**.

The approximation ratio of Algorithm 2 is at least 3.

### *Proof*.

Let *π*=(+3+4−1−2) be a signed permutation. On one hand, we have that Algorithm 2 applies the sequence of signed short reversals *ρ*(2, 4), *ρ*(1, 3), *ρ*(1, 1), *ρ*(2, 2), *ρ*(3, 3), and *ρ*(4, 4) for sorting *π*. On the other hand, we have that the sequence of signed short reversals *ρ*(1, 3) and *ρ*(2, 4) sorts *π*, and the lemma follows.

### **Lemma24**.

The approximation ratio of Algorithm 5 is at least 3.

### *Proof*.

Let *π*=(−3−2−5−4+1) be a signed permutation. On one hand, we have that Algorithm 5 applies the sequence of signed short operations *ρ*(1, 2, 3), *ρ*(3, 4, 5), *ρ*(4, 5), *ρ*(3, 4), *ρ*(2, 3), and *ρ*(1, 2) for sorting *π*. On the other hand, we have that the sequence of signed short operations *ρ*(3, 5) and *ρ*(1, 3) sorts *π*. Therefore the lemma follows.

### **Lemma25**.

The approximation ratio of Algorithm 5 is tight.

### *Proof*.

Immediate from Theorem 5 and Lemma 24.

## Conclusions

In this article, we have presented optimal algorithms for sorting by signed super short reversals and for sorting by signed super short operations, a 5-approximation algorithm for sorting by signed short reversals, and a 3-approximation algorithm for sorting by signed short operations. We have shown that the expected approximation ratio of the 5-approximation algorithm is not greater than 3 for random signed permutations with more than 12 elements. Moreover, the experimental results on small signed permutations have led us to conclude that the approximation ratio of both approximation algorithms cannot be smaller than 3. In particular, this means that the approximation ratio of the 3-approximation algorithm is tight.

We make two remarks. The first remark is that bounding the length of the operations is not the only approach yielded by the assumption that rearrangement events affecting large portions of a genome are less likely to occur. Some researchers [32-34] have proposed to assign weights to the operations according to their length. The second remark is that, as opposed to the unbounded variants of the permutation sorting problem, sorting a linear permutation by short operations is not equivalent to sorting a circular permutation by short operations (see [35] for details). To the best of our knowledge, the only bounded variant considered in the literature that involves circular permutations is the problem of sorting an unsigned circular permutation by reversals of length 2. Jerrum [17] and Egri-Nagy *et al.* [35] demonstrated how to solve this problem in polynomial time.

We see some possible directions for future work. One is to develop polynomial time solutions for the problem of sorting by signed short reversals and for the problem of sorting by signed short operations. Another possibility is to study the problem of sorting signed circular permutations by short operations. In particular, we think that the ideas used to solve the problem of sorting by signed super short reversals can also be used to tackle the problem of sorting a signed circular permutation by reversals of length of at most 2. Finally, one could apply the methods discussed in this work to inferring phylogenies. For instance, Egri-Nagy *et al.* [35] applied their method (*i.e.* sorting unsigned circular permutations by reversals of length 2) to reconstruct the phylogenetic history of some published *Yersinia* genomes. As a result, they produced a phylogenetic tree that is broadly consistent with the phylogenetic tree of Bos *et al.* [36].
